# Maximizing blood pressure lowering effects: a review of drug class comparisons and rationale for combination approaches

**DOI:** 10.3389/fcvm.2025.1656818

**Published:** 2025-11-26

**Authors:** Ying Yang, Feiyan Wan, Jing Xu, Hong Mei Deng, Pei Pan

**Affiliations:** 1 Department of Cardiology, National Center for Cardiovascular Disease, Fuwai Hospital, Beijing, China; 2 Department of Neurology, Guangzhou Eighth People’s Hospital, Guangzhou Medical University, Guangzhou, China; 3 Department of Pharmacy, University-Town Hospital of Chongqing Medical University, Chongqing, China; 4 Department of Neurology, University-Town Hospital of Chongqing Medical University, Chongqing, China; 5 Department of Cardiology, University-Town Hospital of Chongqing Medical University, Chongqing, China

**Keywords:** antihypertensive agents, blood pressure-lowering efficacy, dose-dependent efficacy, combination therapy, hypertension

## Abstract

Numerous studies have reported on the antihypertensive effects of pharmacological treatments, primarily focusing on efficacy comparisons between drug classes, blood pressure (BP)-lowering responses in specific populations, or pleiotropic effects beyond BP reduction. However, the magnitude of BP reduction across agents varies. Additionally, the synergistic effects of combination therapies, and the potential existence of dose-response relationships remain significant clinical dilemmas for physicians. Emerging evidence suggests that many cardiovascular drugs exhibit incidental BP-lowering properties, though further validation is required. Given the critical role of BP management in cardiovascular care coupled with pharmacological heterogeneity and interpatient variability, clinicians face challenges in optimizing targeted treatment strategies for maximal therapeutic benefit. This review synthesizes current evidence on: (1) Drug-class-specific BP-lowering profiles, (2) Dose-dependent efficacy, and (3) Combination therapy strategies. The goal is to provide outcome-driven guidance for clinical decision-making in hypertension management.

## 1 Introduction

Hypertension is the most prevalent chronic disease worldwide and a major risk factor for cardiovascular diseases, stroke, and mortality. According to the latest WHO statistics, approximately 1.4 billion people globally suffer from hypertension, and this number is still growing (1). Current evidence demonstrates that in adults, regardless of age, sex, or the presence of cardiovascular history, diabetes, or other comorbidities, lowering BP is significantly associated with relative and absolute reductions in the risk of cardiovascular events and mortality (2–4). Controlling this risk factor by reducing systolic blood pressure (SBP) by 10, 20, or 30 mmHg to achieve treatment targets, can lower cardiovascular event rates by 29%, 42%, and 54%, respectively (5). Due to variations among patients and antihypertensive medications, studies have found that personalized treatment may reduce SBP by an estimated 4.4 mmHg more than conventional therapy (6), enabling precise BP management. Strong evidence indicates that angiotensin-converting enzyme inhibitors (ACEIs), angiotensin receptor blockers (ARBs), calcium channel blockers (CCBs), diuretics, and β-blockers effectively lower BP and reduce cardiovascular events (7). Other medications, such as alpha-blockers, aldosterone receptor antagonists, potassium-sparing diuretics, angiotensin receptor-neprilysin inhibitors (ARNIs), sodium-glucose cotransporter-2 inhibitors (SGLT2i), and dual endothelin receptor antagonists (DERAs), have also demonstrated antihypertensive effects in clinical practice, though the evidence remains limited.

While the antihypertensive effects of various drug classes are well-documented, questions remain regarding the consistency of dose-response and combination efficacy, blood pressure (BP)-lowering responses in specific populations, or pleiotropic effects beyond BP reduction. However, the magnitude of BP reduction across different antihypertensive agents, the synergistic effects of combination therapies, and the potential existence of dose-response relationships remain significant clinical dilemmas for physicians. Emerging evidence suggests that many cardiovascular drugs exhibit incidental BP-lowering properties, though further validation is required. Given the critical role of BP management in cardiovascular care coupled with pharmacological heterogeneity and interpatient variability, clinicians face challenges in optimizing targeted treatment strategies for maximal therapeutic benefit. This review assesses the effectiveness of commonly antihypertensive drugs in BP-lowering., combination therapies, and certain cardiovascular drugs with antihypertensive properties, based on current evidence. It evaluates the dose-response relationships of these medications and the heterogeneity in BP response during treatment, aiming to address the perplexities faced by cardiovascular physicians and provide better guidance for clinical decision-making.

## 2 Methods

Our study searched the Cochrane Library, PubMed, MEDLINE, Embase, Epistemonikos, and CNKI databases for studies published up to May 31, 2025. Keywords included: Antihypertensive agents, blood pressure-lowering efficacy, dose-dependent efficacy, combination therapy, and hypertension. Publication types encompassed randomized controlled trials, meta-analyses, and systematic reviews. The inclusion criteria were as follows: studies whose primary or key endpoints included the magnitude of blood pressure reduction; scientifically rigorous study designs; well-defined study populations, such as those targeting essential hypertension patients or specific comorbidity groups; and provision of specific blood pressure reduction data. Exclusion criteria consisted of: incomplete data or inability to extract valid blood pressure reduction information; methodologically low-quality studies; and non-Chinese/English publications.

## 3 Evidence efficacy of commonly used antihypertensive drugs

### 3.1 Diuretics

Diuretics exert antihypertensive effects by promoting sodium excretion and reducing volume load. Due to differences in pharmacokinetics and pharmacodynamics, thiazide diuretics are the first-line choice for essential hypertension, while loop diuretics and potassium-sparing diuretics are primarily used in patients with comorbid conditions such as heart failure or chronic kidney disease.

Thiazide diuretics are supported by extensive studies. They not only lower BP but also reduce cardiovascular mortality risk. No clinically significant superiority has been found for other classes of antihypertensive drugs over thiazide diuretics (8–10). Musini et al. (11) conducted a systematic review evaluating the antihypertensive efficacy of six thiazide diuretics in adults with hypertension. The data showed that at the minimum dose required to achieve maximal antihypertensive effect, the mean reductions in systolic/diastolic blood pressure (SBP/DBP) were 9.1/3.6 mmHg. The maximal antihypertensive effect of chlorthalidone (CTDN, 12.5–75 mg/day) was achieved at doses of 12.5–25 mg, with a mean BP reduction of 12.0/3.9 mmHg. Hydrochlorothiazide (HCTZ, 3–100 mg/day) reduced BP by an average of 6.9/3.3 mmHg, 25 mg being the lowest effective dose achieving near-maximal BP reduction (average BP reduction: 8.0/3.3 mmHg). Although higher doses (50–100 mg) showed a greater SBP reduction (>10 mmHg), the difference compared to 25 mg was not statistically significant. Indapamide (NDAP, 1–5 mg/day) reduced BP by an average of 8.7/3.9 mmHg, with the minimum effective dose for optimal antihypertensive effect being 2.5 mg (average reduction: 11.9/5.3 mmHg). While SBP decreased across all baseline groups, DBP reductions varied significantly, especially in the 100–109 mmHg group which showed a greater decline.

While SBP decreased across all baseline groups, DBP reductions varied significantly, especially in the 100–109 mmHg group which showed a greater decline (1.8 mmHg more than other groups), suggesting that NDAP may have a more pronounced effect on DBP reduction. A head-to-head study revealed that NDAP reduced SBP by 5.1 mmHg more than HCTZ, while CTDN achieved a 3.6 mmHg greater SBP reduction compared to HCTZ. These findings suggest that, at equivalent doses, both CTDN and NDAP exhibit superior antihypertensive efficacy over HCTZ (12).

A systematic evaluation of the antihypertensive efficacy of loop diuretics assessed five types and showed that the best estimated BP reduction was 7.9/4.4 mmHg. At equivalent doses, different loop diuretics showed similar antihypertensive effects. Data from the furosemide study indicated: 40 mg/day produced BP reductions of 5.8/3.5 mmHg, 60 mg/day yielded reductions of 10/3 mmHg, While the 60 mg dose showed significant antihypertensive effects, the results carried a high risk of bias, potentially overestimating furosemide's antihypertensive efficacy. Due to scarce dose-response data for loop diuretics in hypertension treatment. However, the analysis was limited by potentially missing BP data and unpublished trails. The antihypertensive efficacy of these agents still requires data validation (13).

Potassium-sparing diuretics are mostly used as second-line antihypertensive agents in combination therapy. Analysis combining data from amiloride (2.5 mg/day) and triamterene (50 mg/day) showed no significant effect on BP reduction (0.03/0.22 mmHg). Low dose amiloride (2.5–5 mg) combined with HCTZ did not show statistically significant differences in BP reduction vs. HCTZ alone. No studies were found evaluating amiloride at doses higher than 5–20 mg/day. It remains unclear whether higher doses provide additional antihypertensive effects, and the dose-response relationship cannot be determined (14).

### 3.2 CCBs

CCBs selectively inhibit voltage-dependent L-type calcium channels on cell membranes, leading to relaxation of vascular smooth muscle and peripheral arterial dilation for BP reduction. They are classified into dihydropyridines and non-dihydropyridines. Wright et al. (15) found that CCBs produced an average BP reduction of 8.9/4.5 mmHg. Amlodipine, a commonly used dihydropyridine CCB in clinical practice, is characterized by: Low renal clearance, long half-life, high bioavailability, absence of rebound hypertension after discontinuation (16). Frick et al. (17) evaluated amlodipine (1.25–10 mg/day) in patients with mild-to-moderate hypertension. The 1.25 mg dose showed no antihypertensive effect compared to placebo, with 2.5 mg being the minimum effective dose. Doses of 5–10 mg reduced BP by 20/9 mmHg vs. placebo. In the CAMELOT study, amlodipine reduced BP by 4.8/2.5 mmHg in coronary artery disease patients with baseline BP of 129/78 mmHg, suggesting its hypotensive effect even in normotensive individuals (18). In the PRAISE-2 trial, amlodipine treatment in heart failure patients (NYHA class III–IV with reduced ejection fraction) resulted in BP reductions of 3.5/2.2 mmHg at 12 weeks, and 2.9/2.1 mmHg at 26 weeks, confirming the safety inpatient with heart failure (19). ACTION study found that nifedipine tablets (30, 60 mg/day) BP decreased by a mean of 5–6/3–4 mmHg (20). Ueng et al. found that nifedipine controlled-release tablets (30, 60 mg/day) decreased overall BP by 27.7/14.1 mmHg, suggesting superior antihypertensive efficacy with long-acting formulations (21). The Syst-Eur study found that patients who received nitrendipine (10–40 mg/day) reduced seated BP by: 10.1/4.5 mmHg at 2 years, 10.7/4.7 mmHg at 4 years (22). ABC Study showed that benidipine (8 mg/day) achieved a BP reduction of 11/9 mmHg (23). Sirri et al. (24) found that both nifedipine controlled-release tablets (30, 60 mg/day) and amlodipine (5, 10 mg/day) significantly lowered BP, but the reductions showed no statistical difference, supporting their clinical equivalence in mild-to-moderate hypertension. A Meta-analysis suggested that there were no significant difference between benidipine and amlodipine in lowering BP (25). Sabbatini et al. found that Lercanidipine can dilate both the renal afferent and efferent arterioles in hypertensive patients (26). Studies have found that lercanidipine demonstrates comparable antihypertensive efficacy to amlodipine, felodipine, and nifedipine sustained-release tablets. Furthermore, lercanidipine prevents renal damage induced by angiotensin II and demonstrates anti-inflammatory, antioxidant, and anti-atherogenic properties through an increasing bioavailability of endothelial nitric oxide (27). While multiple RCTs indicate no significant difference in antihypertensive efficacy between dihydropyridines and non-dihydropyridines, due to the latter pronounced negative inotropic and conductive effects are less commonly used in hypertension (28).

### 3.3 β-Blockers

β-Blockers primarily lower BP by inhibiting the sympathetic nervous system. Guideline recommendations vary across countries (2, 29). Considering their efficacy in conditions with persistent sympathetic overactivation, China recommends β-blockers as first-line antihypertensive therapy. However, their stroke prevention efficacy is weaker than that of other first-line agents. Therefore, ESC guidelines do not recommend them. Meta-analysis shows 9.51/5.64 mmHg BP reduction for all types of β-blockers (15).

A systematic review (30) evaluating the antihypertensive efficacy of non-selective β-blockers, with propranolol and penbutolol as primary data sources, estimated a maximum BP reduction of 10/7 mmHg based on the largest sample sizes (1–2 times the starting dose). After excluding extreme outliers, the reduction was 8/5 mmHg. Within the recommended dose range, no clear dose-response relationship was demonstrated, and higher doses did not yield additional antihypertensive effects.

Systematic review showed that β₁-Blockers lowered BP by an average of 10/8 mmHg, with peak effect (12/9 mmHg) being better than trough effect (8/7 mmHg). Maximum BP lowering was achieved at two times the starting dose, but no graded dose-response relationship was observed within the recommended monotherapy range. Metoprolol (25–400 mg/day) significantly lowered BP in all doses, with 200 mg showing maximum efficacy. No statistical difference in BP reduction between 100 mg and 400 mg. Pooled data (100/200/400 mg): Average reduction of 9/8 mmHg. Bisoprolol (5–20 mg/day): Average reduction of 10/8 mmHg. All doses of atenolol (25–200 mg/day) significantly lowered BP, with the 100 mg showing the greatest BP lowering effect, combined with the data from the 2 subgroups with the largest sample sizes (1 and 2 times the starting dose) yielded reductions of 13/11 mmHg. Atenolol had significantly higher reductions than other β₁-blockers, potentially due to higher baseline BP (162/104 mmHg) in included studies, predominant use of peak BP measurements, possibly amplifying effect size (31).

Wong et al. evaluated the antihypertensive efficacy of dual alpha-beta blockers (32). Including carvedilol (6.25–50 mg/day) and labetalol (400–800 mg/day). The pooled analysis of starting and double doses showed a BP reduction of 6/4 mmHg, with no additional antihypertensive effect observed beyond recommended doses. For carvedilol: 6.25 mg demonstrated no significant antihypertensive effect. Over 12.5 mg elicited significant BP reduction. No clear dose-response relationship was observed within the 12.5–50 mg range. The mean trough BP reduction (pooled starting and double doses) was 4/3 mmHg. For labetalol: 400 mg/day reduced BP by 10.36/6.59 mmHg.800 mg/day showed greater efficacy (19.69/14.58 mmHg). The lack of data on the recommended starting dose (200 mg/day), and the small sample sizes, may lead to biased studies and overestimation of antihypertensive effects.

### 3.4 ACEIs

By dually targeting RASS and kinin pathways, ACEIs achieve comprehensive antihypertensive effects. Meta-analysis of trough antihypertensive effects of 14 ACEIs (33) and it was found that there is no statistically significant difference in BP-lowering effects among different ACEIs. At half to maximum (Max) recommended dose, BP decreased by 8/5 mmHg, and the average peak BP decreased by 11/6 mmHg. The 1/16 Max recommended dose has no measurable antihypertensive effect. The recommended starting dose (1/8 or 1/4 Max) can achieve 60%–70% of Max therapeutic effect. 1/2 Max recommended dose can achieve approximately 90% of the maximum therapeutic effect. Exceeding the Max recommended dose does not have additional antihypertensive benefit demonstrated. In evaluations of benazepril (2–80 mg/day), doses of 2–10 mg did not significantly reduce BP. A 20 mg dose was the minimum effective dose, and 20–80 mg achieved near-maximal effect (8.70/4.92 mmHg reduction). Captopril (37.5–200 mg/day) had an estimated near-maximal lowering effect of 9.68/5.43 mmHg. Enalapril (5–20 mg/day) had a near-maximal lowering effect (8.66/4.80 mmHg reduction). The lowest effective dose was 5 mg, it is possible that an antihypertensive effect exists at lower doses, but lack of data to support this. Fosinopril (2.5–40 mg/day): 2.5 mg and 5 mg showed no significant antihypertensive effect, 20 mg and 40 mg produced statistically significant BP reduction, with the lowest effective dose ranging from 10 to 20 mg and an estimated reduction of 7.62/5.00 mmHg. Lisinopril (1.25–80 mg/day) demonstrated significant reductions in BP from10–80 mg, with reductions of 8.00/4.76 mmHg, 10 mg was the lowest dose with near-maximal antihypertensive effect, insufficient data for <10 mg, lowest effective dose unknown, and no significant difference in antihypertensive effect when comparing 10 mg, 20 mg, and 80 mg. Perindopril (2–16 mg/day) showed no antihypertensive effect at 2 mg, the lowest effective dose was 4 mg, and 4–16 mg dose range produced a reduction of 7.09/5.02 mmHg, with limited data in the high dose region (8, 16 mg) to confirm the dose-response. Ramipril (1.25–10 mg/day) showed no significant antihypertensive effect with 1.25–2.5 mg, the lowest effective dose was 5 mg, and the optimal range of antihypertensive doses was 5–10 mg (mean reduction: 6.29/4.14 mmHg), with no additional benefit from escalation to 10 mg/day.

### 3.5 ARBs

By selectively antagonizing the Ang II AT1 receptor, ARBs induce vasodilation, reduce fluid retention, and dampen sympathetic overactivity, collectively contributing to their antihypertensive efficacy. Heran et al. systematically reviewed the trough BP received with different ARBs, suggesting that the antihypertensive effects of different ARBs were similar, and the magnitude is comparable to that of ACEls. The recommended starting dose (1/8–1/4 Max recommended dose) could achieve 60%–70% of the Max effect, and the 1/2 Max recommended dose reached 80% of the Max recommended dose. The reduction of BP was 6–10/3–7 mmHg. When the dose was greater than the recommended Max, there was no significant antihypertensive effect. At the maximum and higher doses, the BP reduction was 9.0/5.6 mmHg, and the average peak BP decreased by 12.0/7.0 mmHg (34). The evaluation of candesartan (2–32 mg/day) demonstrated: 2 mg produced no meaningful reduction in BP; The minimum effective dose was 4 mg, achieving nearly 90% of the maximum antihypertensive effect; The optimal reduction (4–32 mg range) was 8.93/5.59 mmHg, but no statistically significant dose-response relationship was observed. The evaluation of irbesartan (37.5–300 mg/day) indicated that doses above 50 mg demonstrated significant BP reduction; No statistically significant differences were observed between 75 and 300 mg doses, with an average reduction of 7.91/5.09 mmHg. The evaluation of losartan (10–150 mg/day) demonstrated: 10 mg and 25 mg did not produce clinically significant BP reduction; 50 mg was the lowest dose achieving near-maximal antihypertensive efficacy; The maximum BP reduction (50–150 mg) was 6.64/3.59 mmHg. Olmesartan (10–40 mg/day) evaluation demonstrated 20 mg was the minimum effective dose; The maximal BP reduction (20–40 mg) was 10.39/7.31 mmHg. Evaluation of telmisartan (20–160 mg/day) demonstrated minimum effective dose was 20 mg; Maximum BP reduction (20–160 mg dose range) was 8.38/6.69 mmHg. Valsartan (10–320 mg/day) evaluation results:10 mg dose did not observable antihypertensive effect; The minimum effective dose was 20 mg;80 mg was the lowest dose achieving near-maximal antihypertensive efficacy; 80–320 mg dosage range achieved a BP reduction of 7.10/4.34 mmHg, with limited antihypertensive value of incremental increases to >80 mg.

### 3.6 α-Adrenergic blockers

α-adrenergic blockers lower BP by selectively blocking α1-adrenergic receptors on vascular smooth muscle, leading to vasodilation and reduced peripheral vascular resistance. Heran et al. evaluated the effect of different α-adrenergic blockers troughs in lowering BP and showed similar reductions, with a best estimate of 8/5 mmHg. Doxazosin (2–12 mg/day) at 4 mg achieved an average BP reduction of 6.42/3.53 mmHg. Prazosin (2.5–20 mg/day) evaluation demonstrated: Low dose (2.5–3 mg) did not produce a significant antihypertensive effect; 10 mg was the minimum effective dose; 20 mg vs. 10 mg observed no additional statistical antihypertensive effect; Optimal reduction (10–20 mg) was 10.38/6.90 mmHg. The evaluation of terazosin (5–20 mg/day) demonstrated: 5 mg (possibly lower, but data limited) was the minimum effective dose; 10 mg and 20 mg significantly lowered BP, but did not show a inter-dose statistical difference, and the mean reduction across all dose data was 6.59/4.40 mmHg (35).

### 3.7 Direct renin inhibitors

Direct renin inhibitors exert their antihypertensive effect by suppressing renin activity, thereby reducing Angiotensin I production and subsequently lowering Angiotensin II and aldosterone levels. Musini et al. (36) showed that aliskiren 75, 150, 300, and 600 mg/day lowered BP by 2.6/2.1, 5.6/2.9.7.9/4.8, and 11.4/6.6 mmHg compared to placebo, with 150–600 mg averaging an 8.30/4.76 mmHg reduction; 300 mg vs. 150 mg significantly enhanced antihypertensive efficacy.

## 4 Novel antihypertensive therapies awaiting cardiovascular outcome trial evidence

Research has identified that certain medications primarily indicated for heart failure and diabetes—including ARNIs, SGLT2i, aldosterone synthase inhibitors, and dual endothelin receptor antagonists demonstrate secondary antihypertensive effects. Masash et al. (37) provided a comprehensive rationale for advancing the clinical development of soluble guanylate cyclase stimulators and activators for the treatment of hypertension. However, broader application for hypertension management requires verification through dedicated cardiovascular outcome trials before consideration in treatment algorithms (2).

### 4.1 ARNIs

ARNIs achieves synergistic BP lowering by enhancing the natriuretic peptide system and blocking renin-angiotensin system (RAAS). Kario et al. found that sacubitril/valsartan at doses of 100 mg, 200 mg, and 400 mg/day reduced Clinic BP by 11.86/7.84 mmHg, 12.57/7.29 mmHg, and 15.38/8.76 mmHg respectively compared to placebo, demonstrating excellent efficacy in Asian hypertensive patients (38). Meta-analysis showed sacubitril/valsartan at 100 mg, 200 mg, and 400 mg reduced SBP by an average of 8.94 mmHg, 11.77 mmHg, and 14.20 mmHg respectively vs. placebo (39). De Vecchis et al. (40) found elderly hypertensive patients receiving ARNI had average seated BP reductions of 19.53/8.33 mmHg and ambulatory BP reductions of 13.34/7.25 mmHg, showing antihypertensive efficacy in elderly patients.

### 4.2 Aldosterone receptor antagonists

Aldosterone receptor antagonists lower BP by blocking aldosterone receptor binding, reducing sodium and water retention, and suppressing sympathetic activity and RAAS feedback activation. A meta-analysis demonstrated that spironolactone (25–500 mg/day) reduced BP by an average of 20.09/6.75 mmHg. While the 25 mg dose showed no statistically significant antihypertensive effect, doses of 100–500 mg produced significant BP reduction, though no clear dose-response relationship was observed (41). Tam et al. assessed eplerenone (25–400 mg/day) showing a reduction of 9.21/4.18 with 50–200 mg mmHg, with no definitive antihypertensive effect at 25 mg and insufficient evidence at doses above 200 mg. Weinberger et al. (42) found that eplerenone 400 mg/day produced an average SBP reduction of 16.5 mmHg, with non-overlapping confidence intervals compared to other doses, suggesting a possible quantitative efficacy relationship at doses of 400 mg to higher doses, but there is a lack of studies confirming this. The PATHWAY-2 study demonstrated that in patients whose BP remained uncontrolled after ≥3 months on three maximally tolerated antihypertensive agents, add-on spironolactone (25–50 mg/day) achieved 8.70 mmHg greater home SBP reduction vs. placebo (43). In the BrigHTN study (patients already on three antihypertensive drugs), the novel aldosterone synthase inhibitor baxdrostat (administered once daily at 0.5 mg, 1 mg, or 2 mg) showed additional clinic seated SBP reductions of 2.7 mmHg, 8.1 mmHg, and 11.0 mmHg respectively compared to placebo (44).

### 4.3 DERAs

DERAs reduce BP by blocking both endothelin type A(ET-A) and type B(ET-B)receptors. Aprocitentan is currently the only DERA approved for the treatment of resistant hypertension in adults. Pierre et al. (45) found that aprocitentan at doses of 10, 25, and 50 mg/day reduced seated BP by 7.05/4.93, 9.90/6.99, and 7.58/4.95 mmHg, respectively, compared to placebo. The 5 mg dose showed no statistically significant difference vs. placebo. Similar advantages were observed in patients with valid ambulatory blood pressure monitoring data.

### 4.4 SGLT2i

SGLT2i have demonstrated cardiovascular event and renal hemodynamic benefits in clinical trials involving patients with type 2 diabetes and heart failure, with additional antihypertensive effects observed. A meta-analysis showed a mean BP reduction of 4.0/1.6 mmHg over placebo (46). Mazidi et al. (47) found that SGLT2i have significant reduction in BP (2.46/1.46 mmHg). Wever et al. reported a > 10% SBP reduction with losartan-empagliflozin combination therapy vs. losartan monotherapy (48). A synergistic antihypertensive effect may exist.

## 5 Differential blood pressure reduction by different antihypertensive agents

Summary of antihypertensive efficacy evidence (Table 1), the first-line five major classes of drugs lowered BP similarly, with a mean reduction of 9.1/5.5 mmHg for the recommended dose and 7.1/4.4 mmHg for the half-dose (It dropped by 20%). ARNIs demonstrate superior BP-lowering effects, and aldosterone receptor antagonists showed good results in the treatment of refractory hypertension. Some studies provide evidence of heterogeneity in response to hypertension drug therapy, but the differences are small (6). The fundamental reason might be the differences in pharmacodynamics and mechanism of action. A systematic review suggested that thiazide diuretics SBP decreased 1.36–3.01 mmHg more than β-Blockers, CCBs, ACEls, α-adrenergic blockers, and ARBs, and 0.90 mmHg less than direct renin inhibitors. Except for DBP, which was elevated by 0.96 mmHg compared to CCBs, and the difference was not statistically significant compared to the other classes (10). Zhu et al. found that CCBs reduced SBP by 1.11 mmHg and 2.10 mmHg more than ACEIs and ARBs, and by 0.81 mmHg less than diuretics. DBP decreased by 0.63–1.70 mmHg more than diuretics, ACEIs, ARBs and α-adrenergic blockers, and there was no significant difference in SBP/DBP compared with β-blockers (49). Wang et al. (50) found that DERAs reduced SBP by 1.72 mmHg and DBP by 1.18 mmHg more than ACEIs. Meta-analysis showed an additional 5–7 mmHg reduction in mean SBP for ARNls compared to ARBs (39, 51). The PATHWAY-2 study found that patients with refractory hypertension receiving spironolactone had an additional SBP reduction of 4.03 and 4.48 mmHg compared todoxazosin and bisoprolol (43). The antihypertensive effect of dual alpha-beta blockers is slightly weaker than that of non-selective β-blockers, β1 receptor blockers, thiazides, ACEIs, and ARBs (15).

**Table 1:** Blood pressure-lowering effects of different antihypertensive agents.

Category of drug	Drug (daily dose)	Mean BP reduction	Maximal BP reduction	Cumulative BP reduction	BP reduction at specified doses	Refs.
		SBP/DBP mmHg				
Diuretics						
Thiazide diuretics			9.1/3.6			
	CTDN (12.5–75 mg)	6.9/3.3	12.0/3.9 (12.5–25 mg)			Musini et al. (11)
	HCTZ (3–100 mg)	8.7/3.9	8.0/3.3 (25 mg)			
	NDAP (1–5 mg)	7.9/4.4	11.9/5.3 (2.5 mg)			
Loop diuretics						
	Furosemide (40–60 mg)				5.80/3.53 (40 mg)	Musini et al. (13)
					10/3 (60 mg)	
Potassium-sparing diuretics						
	Amiloride (2.5–5 mg)			0.03/0.22 (amiloride 2.5 mg+triamterene 50 mg)		Heran et al. (14)
	Triamterene (50 mg)					
CCBs		8.9/4.5				
	Amlodipine (1.25–10 mg)			20/9 (5–10 mg)		Wright and Musini (15)
	Nifedipine (30–60 mg)	5–6/3–4				Frick et al. (17)
	Nifedipine controlled-release (30–60 mg)	27.7/14.1				Poole-Wilson et al. (20)
	Nitrendipine (10–40 mg)	10.1/4.5 (follow-up 2 year)				Ueng et al. (21)
		10.7/4.7 (follow-up 2 year)				Staessen et al. (22)
	Benidipine (8 mg)	11.0/9.0				Ohishi et al. (23)
ACEIs		8/5 (trough)				
		11/6 (peak)				
	Benazepril (2–80 mg)				8.70/4.92 (20 mg)	
	Enalapril (5–20 mg)				8.66/4.80 (2 mg)	
	Captopril (37.5–200 mg)	9.68/5.43				Heran et al. (33)
	Fosinopril (2.5–40 mg)			7.62/5.00 (10–20 mg)		
	Lisinopril (1.25–80 mg)				8.00/4.76 (10 mg)	
	Perindopril (2–16 mg)			7.09/5.02 (4–16 mg)		
	Ramipril (1.25–10 mg)			6.29/4.14 (5–10 mg)		
ARBs		6–10/3–7				
	Candesartan (12–32 mg)			8.93/5.59 (4–32 mg)		
	Irbesartan (37.5–300 mg)		7.91/5.09 (75–300 mg)			
	Losartan (10–150 mg)		6.64/3.59 (50–150 mg)			Heran et al. (34)
	Olmesartan (10–40 mg)		10.39/7.31 (20–40 mg)			
	Telmisartan (20–160 mg)		8.38/6.69 (20–160 mg)			
	Valsartan (10–320 mg)		7.10/4.34 (80–320 mg)			
β-Blockers		9.51/5.64				Wright and Musini (15)
Non-selective β-Blockers		8.0/5.0				Wong et al. (30)
	Propranolol (80–320 mg)					
β₁-Blockers		10.0/8.0		9.0/8.0 (100–400 mg)		
	Metoprolol (25–400 mg)					Wong et al. (31)
	Bisoprolol (5–20 mg)	10.0/8.0				
	Atenolol (25–200 mg)			13.0/11.0 (5–100 mg)		
Dual alpha-beta blockers				6.0/4.0 (starting and double doses)		
	Carvedilol (6.25–50 mg)			4.0/3.0 (12.5/25 mg)		Wong et al. (32)
	Labetalol (400–800 mg)				10.36/6.59 (400 mg)	
					19.69/14.58 (800 mg)	
α-Adrenergic blockers			6.42/3.53			
	Doxazosin (2–12 mg)		10.38/6.90 (10–20 mg)			Heran et al. (35)
	Prazosin (2.5–20 mg)	8.0/5.0				
	Terazosin (5–20 mg)		6.59/4.40 (5–20 mg)			
Direct renin inhibitors						
	Aliskiren (75–600 mg)			8.30/4.76 (150–600 mg)		Musini et al. (36)
Aldosterone receptor antagonists						
	Spironolactone (25–500 mg)	20.09/6.75		8.70/– (25–50 mg)		Batterink et al. (41)
	Eplerenone (25–400 mg)			9.21/4.18 (50–200 mg)		Tam et al. (42)
	Baxdrostat (0.5–2 mg)			9.55/– (1–2 mg)		Awosika et al. (44)
ARNI						
	Sacubitril/valsartan (100–400 mg)				11.86/7.84 (100 mg)	
					12.57/7.29 (200 mg)	Kario et al. (38)
					15.38/8.76 (400 mg)	
SGLT2-i						
	Canagliflozin (50–600 mg)					
	Dapagliflozin (1–10 mg)					
	Empagliflozin (1–50 mg)	4.0/1.6				Baker et al. (46)
	Ipragliflozin (12.5–300 mg)					
	Remogliflozin (200–1,000 mg)					
DERAs						
	Aprocitentan (10–50 mg)	8.17/5.62				Verweij et al. (45)

## 6 Evidence and trends in combination therapy

Combination of different drug classes can produce additive or synergistic effects, achieving significantly greater BP reduction compared to dose escalation of a single agent. This is partly attributed to simultaneous modulation of multiple pathophysiological mechanisms while reducing adverse effects through lower individual drug doses. For most hypertensive patients, combination therapy is necessary. The BPLTTC study demonstrated that the actual BP reduction from dual-drug combinations closely aligns with the theoretical additive effect of individual drugs—the observed efficacy of dual-drug therapy was nearly equivalent to the sum of their individual effects (52). David S et al. (53) also confirmed that the expected reduction in BP closely matched the actual measured values, with the additional antihypertensive effect of combination therapy being approximately five times greater than doubling the dose of a single drug. The 2015 Taiwan hypertension guidelines introduced the “Rule of 10 and 5” for predicting the reduction in SBP/DBP with either monotherapy or combination therapy. Specifically, when baseline BP is 154/97 mmHg, standard-dose treatment with any of the five major drug classes is expected to lower SBP by 10 mmHg and DBP by 5 mmHg (54). For every 10 mmHg increase in baseline BP, an additional reduction of 1.0/1.1 mmHg can be anticipated. Based on the projected BP reduction from monotherapy and dual therapy, triple therapy is expected to achieve a reduction of 20/11 mmHg (55).

The meta-analysis showed that compared to monotherapy/conventional treatment, low-dose triple therapy achieved: Greater SBP reduction after 4–12 weeks (7.4 mmHg additional reduction), 18.0 mmHg greater reduction vs. placebo; Higher target achievement rate (66% vs. 46%);Sustained SBP reduction advantage of approximately 6.4 mmHg over monotherapy/conventional treatment during 6–12 months follow-up (56). Rodgers et al. (57) evaluated the efficacy of GMRx2 (a low-dose combination of telmisartan, amlodipine, and NDAP) vs. dual therapy. The study demonstrated that GMRx2 achieved significantly greater reductions in office BP compared to dual therapy, ranging from 4.3–6.3 mmHg SBP and 3.5–4.5 mmHg DBP. Notably, the antihypertensive advantage was similar between half-dose and standard-dose regimens, with an average reduction of 5.1/3.7 mmHg. The QUARTET study evaluated the antihypertensive efficacy of a quadpill (four-drug combination) vs. monotherapy. Results demonstrated superior BP reduction with the quadpill: At 12 weeks, the quadpill demonstrated significantly greater BP reduction compared to monotherapy (6.9/5.8 mmHg); At 52 weeks with 7.7/6.0 mmHg greater BP reduction; Additionally, the quadpill showed significantly higher BP control rates throughout the study period (58). Current evidence demonstrates promising antihypertensive efficacy for both standard-dose and quarter-dose quadpill regimens, though the precise magnitude of BP reduction requires further investigation through evidence-based medical research (53, 59). The meta-analysis demonstrated that fixed-dose combination (FDC) therapy significantly improved medication adherence compared to triple combinations, while showing comparable efficacy in BP reduction and adverse effect profiles (60, 61). While FDC therapy reduced clinical inertia and achieved faster time to BP target than conventional treatment (62, 63).

## 7 Conclusion

Regarding whether there are differences in antihypertensive effects among first-line drugs, current studies have confirmed similar efficacy across drug classes: standard doses achieve an average BP reduction of 9.1/5.5 mmHg, while half doses reduce BP by 7.1/4.4 mmHg; 1/8–1/4 of the maximum recommended dose reaches the minimum effective dose, 1/2 maximum dose achieves 70%–80% of maximal antihypertensive efficacy, and double the starting dose attains the maximum blood pressure-lowering effect. Most studies have not identified a clear dose-response relationship for antihypertensive drugs, with higher doses failing to produce greater BP reduction while increasing adverse effects. Current research still cannot fully explain the differences in antihypertensive efficacy between some drugs, which may be fundamentally attributed to the utilization of self-blood pressure monitoring (64), variations in pharmacodynamics, pharmacokinetics, and drug-related genes. Differences in heterogeneous measurement methods across studies and the resulting lack of comparability may also contribute. Most drugs lack broad dose-range data, necessitating head-to-head trials to verify potential differences at equivalent doses and provide reference for dose equivalency. Due to variations in clinical BP measurement methods, specific measurement timing (peak/trough values), and baseline BP, routine clinical measurements may differ from actual therapeutic effects. ARNIs demonstrate superior antihypertensive effects compared to first-line antihypertensive drugs and may potentially become commonly treatments for hypertension. Given the antihypertensive efficacy and personalized potential of combination therapy, low-dose triple and quadruple combinations may emerge as potential initial treatment strategies for hypertension.

Our study may have limitations in search comprehensiveness. Additionally, due to the varying quality of included studies, we did not employ statistical methods to fully quantify the magnitude of blood pressure reduction. Future research should incorporate high-quality studies to thoroughly quantify the blood pressure-lowering effects of various antihypertensive agents and evaluate whether these effects differ across disease states and baseline blood pressure levels. Further investigation into the non-blood pressure-lowering cardiovascular protective mechanisms of antihypertensive drugs is warranted to provide additional evidence for clinical decision-making.

## References

[1] Organization Geneva: World Health. Global Report on Hypertension: The Race Against a Silent Killer. Geneva: World Health Organization (2023).

[2] McEvoy JW McCarthy CP Bruno RM Brouwers S Canavan MD Ceconi C 2024 ESC guidelines for the management of elevated blood pressure and hypertension. Eur Heart J. (2024) 45(38):3912–4018. 10.1093/eurheartj/ehae178 39210715

[3] Blood Pressure Lowering Treatment Trialists’ Collaboration. Pharmacological blood pressure lowering for primary and secondary prevention of cardiovascular disease across different levels of blood pressure: an individual participant-level data meta-analysis. Lancet. (2021) 397(10285):1625–36. 10.1016/S0140-6736(21)00590-0 33933205 PMC8102467

[4] Fuchs FD Fuchs SC Berwanger O Whelton PK. Clinical trials in hypertension: a mathematical endorsement for diagnosis and treatment. Hypertension. (2025) 82(3):411–8. 10.1161/HYPERTENSIONAHA.124.21361 39970255 PMC11841924

[5] Carey Robert M Moran Andrew E Whelton Paul K. Treatment of hypertension: a review. JAMA. (2022) 328(18):1849–61. 10.1001/jama.2022.19590 36346411

[6] Sundström J Lind L Nowrouzi S Hagström E Held C Lytsy P Heterogeneity in blood pressure response to 4 antihypertensive drugs: a randomized clinical trial. JAMA. (2023) 329(14):1160–9. 10.1001/jama.2023.3322 37039792 PMC10091169

[7] Yamal J-M Martinez J Osani MC Du XL Simpson LM Davis BR. Mortality and morbidity among individuals with hypertension receiving a diuretic, ACE inhibitor, or calcium channel blocker: a secondary analysis of a randomized clinical trial. JAMA Netw Open. (2023) 6(12):e2344998. 10.1001/jamanetworkopen.2023.44998 38048133 PMC10696481

[8] Wei J Galaviz KI Kowalski AJ Magee MJ Haw JS Narayan KMV Comparison of cardiovascular events among users of different classes of antihypertension medications: a systematic review and network meta-analysis. JAMA Netw Open. (2020) 3(2):e1921618. 10.1001/jamanetworkopen.2019.21618 32083689 PMC7043193

[9] Thomopoulos C Parati G Zanchetti A. Effects of blood pressure-lowering on outcome incidence in hypertension: 5. Head-to-head comparisons of various classes of antihypertensive drugs—overview and meta-analyses. J Hypertens. (2015) 33(7):1321–41. 10.1097/HJH.0000000000000614 26039526

[10] Reinhart M Puil L Salzwedel DM Wright JM. First-line diuretics versus other classes of antihypertensive drugs for hypertension. Cochrane Database Syst Rev. (2023) 7(7):CD008161. 10.1002/14651858.CD008161.pub3 37439548 PMC10339786

[11] Musini VM Nazer M Bassett K Wright JM. Blood pressure-lowering efficacy of monotherapy with thiazide diuretics for primary hypertension. Cochrane Database Syst Rev. (2014) 2014(5):CD003824. 10.1002/14651858.CD003824.pub2 24869750 PMC10612990

[12] Roush GC Ernst ME Kostis JB Tandon S Sica DA. Head-to-head comparisons of hydrochlorothiazide with indapamide and chlorthalidone: antihypertensive and metabolic effects. Hypertension. (2015) 65(5):1041–6. 10.1161/HYPERTENSIONAHA.114.05021 25733245

[13] Musini VM Rezapour P Wright JM Bassett K Jauca CD. Blood pressure-lowering efficacy of loop diuretics for primary hypertension. Cochrane Database Syst Rev. (2015) 2015(5):CD003825. 10.1002/14651858.CD003825.pub4 26000442 PMC7156893

[14] Heran BS Chen JM Wang JJ Wright JM. Blood pressure lowering efficacy of potassium-sparing diuretics (that block the epithelial sodium channel) for primary hypertension. Cochrane Database Syst Rev. (2012) 11(11):CD008167. 10.1002/14651858.CD008167.pub2 23152254 PMC11380160

[15] Wright JM Musini VM. First-line drugs for hypertension. Cochrane Database Syst Rev. (2018) 4(4):CD001841. 10.1002/14651858.CD001841.pub2 29667175 PMC6513559

[16] Wang J Palmer BF Vogel Anderson K Sever P. Amlodipine in the current management of hypertension. J Clin Hypertens. (2023) 25(9):801–7. 10.1111/jch.14709 PMC10497034 37551050

[17] Frick MH McGibney D Tyler HM. Amlodipine: a double-blind evaluation of the dose-response relationship in mild to moderate hypertension. J Cardiovasc Pharmacol. (1988) 12(7):S76–8. 10.1097/00005344-198812007-00017 2467135

[18] Nissen SE Tuzcu EM Libby P Thompson PD Ghali M Garza D Effect of antihypertensive agents on cardiovascular events in patients with coronary disease and normal blood pressure: the CAMELOT study: a randomized controlled trial. JAMA. (2004) 292(18):2217–25. 10.1001/jama.292.18.2217 15536108

[19] Packer M Carson P Elkayam U Konstam MA Moe G O'Connor C Effect of amlodipine on the survival of patients with severe chronic heart failure due to a nonischemic cardiomyopathy: results of the PRAISE-2 study (prospective randomized amlodipine survival evaluation 2). JACC Heart Fail. (2013) 1(4):308–14. 10.1016/j.jchf.2013.04.004 24621933

[20] Poole-Wilson PA Lubsen J Kirwan B-A van Dalen FJ Wagener G Danchin N Effect of long-acting nifedipine on mortality and cardiovascular morbidity in patients with stable angina requiring treatment (ACTION trial): randomised controlled trial. Lancet. (2004) 364(9437):849–57. 10.1016/S0140-6736(04)16980-8 15351192

[21] Ueng K-C Ningling S El Maksod A Hung K-Y Yuehui Y. Efficacy and tolerability of long-acting nifedipine GITS/OROS monotherapy or combination therapy in hypertensive patients: results of a 12-week international, prospective, multicentre, observational study. Clin Drug Investig. (2011) 31(9):631–42. 10.2165/11588970-000000000-00000 21591818

[22] Staessen JA Fagard R Thijs L Celis H Arabidze GG Birkenhäger WH Randomised double-blind comparison of placebo and active treatment for older patients with isolated systolic hypertension. The systolic hypertension in Europe (Syst-Eur) trial investigators. Lancet. (1997) 350(9080):757–64. 10.1016/S0140-6736(97)05381-6 9297994

[23] Ohishi M Takagi T Ito N Terai M Tatara Y Hayashi N Renal-protective effect of T-and L-type calcium channel blockers in hypertensive patients: an amlodipine-to-benidipine changeover (ABC) study. Hypertens Res. (2007) 30(9):797–806. 10.1291/hypres.30.797 18037772

[24] Kes S Caglar N Canberk A Deger N Demirtas M Dortlemez H Treatment of mild-to-moderate hypertension with calcium channel blockers: a multicentre comparison of once-daily nifedipine GITS with once-daily amlodipine. Curr Med Res Opin. (2003) 19(3):226–37. 10.1185/030079903125001677 12803737

[25] Sofy AA Abdelsattar AT Mohammed OM Shareef MA Alamodi AA Nso N Amlodipine compared with benidipine in the management of hypertension: a systematic review and meta-analysis. High Blood Press Cardiovasc Prev. (2020) 27(6):527–37. 10.1007/s40292-020-00412-y 33001356

[26] Sabbatini M Leonardi A Testa R Vitaioli L Amenta F. Effect of calcium antagonists on glomerular arterioles in spontaneously hypertensive rats. Hypertension. (2000) 35(3):775–9. 10.1161/01.HYP.35.3.775 10720594

[27] Grassi G Robles NR Seravalle G Fici F. Lercanidipine in the management of hypertension: an update. J Pharmacol Pharmacother. (2017) 8(4):155–65. 10.4103/jpp.JPP_34_17 29472747 PMC5820745

[28] Pepine CJ Handberg EM Cooper-DeHoff RM Marks RG Kowey P Messerli FH A calcium antagonist vs a non-calcium antagonist hypertension treatment strategy for patients with coronary artery disease. The international verapamil-trandolapril study (INVEST): a randomized controlled trial. JAMA. (2003) 290(21):2805–16. 10.1001/jama.290.21.2805 14657064

[29] Revision Committee of Chinese Guidelines for Hypertension Prevention and Treatment, Hypertension Alliance (China), Hypertension Branch of China International Exchange and Promotive Association for Medical and Health Care, Task Force of the Chinese Hypertension Guidelines, Chinese Hypertension League, Hypertension Branch of the China International Exchange and Promotive Association for Medical and Health Care, et al. Chinese Guidelines for the prevention and treatment of hypertension (2024 revision). Chin J Hypertens. (2024) 32(7):603–700. 10.26599/1671-5411.2025.01.008

[30] Wong Gavin WK Wright James M. Blood pressure lowering efficacy of nonselective beta-blockers for primary hypertension. Cochrane Database Syst Rev. (2014) 2014(2):CD007452. 10.1002/14651858.CD007452.pub2 24585007 PMC10603273

[31] Wong GW Boyda HN Wright JM. Blood pressure lowering efficacy of beta-1 selective beta blockers for primary hypertension. Cochrane Database Syst Rev. (2016) 3(3):CD007451. 10.1002/14651858.CD007451.pub2 26961574 PMC6486283

[32] Wong GW Laugerotte A Wright JM. Blood pressure lowering efficacy of dual alpha and beta blockers for primary hypertension. Cochrane Database Syst Rev. (2015) 2015(8):CD007449. 10.1002/14651858.CD007449.pub2 26306578 PMC6486308

[33] Heran BS Wong MM Heran IK Wright JM. Blood pressure lowering efficacy of angiotensin converting enzyme (ACE) inhibitors for primary hypertension. Cochrane Database Syst Rev. (2008) 2008(4):CD003823. 10.1002/14651858.CD003823.pub2 18843651 PMC7156914

[34] Heran BS Wong MM Heran IK Wright JM. Blood pressure lowering efficacy of angiotensin receptor blockers for primary hypertension. Cochrane Database Syst Rev. (2008) 2008(4):CD003822. 10.1002/14651858.CD003822.pub2 18843650 PMC6669255

[35] Heran BS Galm BP Wright JM. Blood pressure lowering efficacy of alpha blockers for primary hypertension. Cochrane Database Syst Rev. (2012) 2012(8):CD004643. 10.1002/14651858.CD004643.pub3 22895943 PMC11337167

[36] Musini VM Lawrence KA Fortin PM Bassett K Wright JM. Blood pressure lowering efficacy of renin inhibitors for primary hypertension. Cochrane Database Syst Rev. (2008) 4(4):CD007066. 10.1002/14651858.CD007066.pub3 18843743

[37] Tawa M Nakagawa K Ohkita M. Soluble guanylate cyclase stimulators and activators as potential antihypertensive drugs. Hypertens Res. (2025) 48(4):1458–70. 10.1038/s41440-025-02110-5 39833553

[38] Kario K Sun N Chiang F-T Supasyndh O Baek SH Inubushi-Molessa A Efficacy and safety of LCZ696, a first-in-class angiotensin receptor neprilysin inhibitor, in Asian patients with hypertension: a randomized, double-blind, placebo-controlled study. Hypertension. (2014) 63(4):698–705. 10.1161/HYPERTENSIONAHA.113.02002 24446062

[39] Malik AH Aronow WS. Efficacy of sacubitril/valsartan in hypertension. Am J Ther. (2022) 29(3):e322–33. 10.1097/MJT.0000000000000925 30664018

[40] De Vecchis R Soreca S Ariano C. Anti-hypertensive effect of sacubitril/valsartan: a meta-analysis of randomized controlled trials. Cardiol Res. (2019) 10(1):24–33. 10.14740/cr813 30834056 PMC6396804

[41] Batterink J Stabler SN Tejani AM Fowkes CT. Spironolactone for hypertension. Cochrane Database Syst Rev. (2010) (8):CD008169. 10.1002/14651858.CD008169.pub2 20687095

[42] Tam TS Wu MH Masson SC Tsang MP Stabler SN Kinkade A Eplerenone for hypertension. Cochrane Database Syst Rev. (2017) 2(2):CD008996. 10.1002/14651858.CD008996.pub2 28245343 PMC6464701

[43] Williams B MacDonald TM Morant S Webb DJ Sever P McInnes G Spironolactone versus placebo, bisoprolol, and doxazosin to determine the optimal treatment for drug-resistant hypertension (PATHWAY-2): a randomised, double-blind, crossover trial. Lancet. (2015) 386(10008):2059–68. 10.1016/S0140-6736(15)00257-3 26414968 PMC4655321

[44] Awosika A Cho Y Bose U Omole AE Adabanya U. Evaluating phase II results of Baxdrostat, an aldosterone synthase inhibitor for hypertension. Expert Opin Investig Drugs. (2023) 32(11):985–95. 10.1080/13543784.2023.2276755 37883217

[45] Verweij P Danaietash P Flamion B Ménard J Bellet M. Randomized dose-response study of the new dual endothelin receptor antagonist aprocitentan in hypertension. Hypertension. (2020) 75(4):956–65. 10.1161/HYPERTENSIONAHA.119.14504 32063059 PMC7098434

[46] Baker WL Smyth LR Riche DM Bourret EM Chamberlin KW White WB. Effects of sodium-glucose co-transporter 2 inhibitors on blood pressure: a systematic review and meta-analysis. J Am Soc Hypertens. (2014) 8(4):262–75.e9. 10.1016/j.jash.2014.01.007 24602971

[47] Mazidi M Rezaie P Gao H Kengne AP. Effect of sodium-glucose cotransport-2 inhibitors on blood pressure in people with type 2 diabetes mellitus: a systematic review and meta-analysis of 43 randomized control trials with 22 528 patients. J Am Heart Assoc. (2017) 6(6):e004007. 10.1161/JAHA.116.004007 28546454 PMC5669140

[48] Wever BE Scholtes RA Mosterd CM Hesp AC Smits MM Heerspink HJL The systemic and kidney hemodynamic response to empagliflozin, losartan and their combination varies between individuals. J Nephrol. (2025) 38(6):1647–53. 10.1007/s40620-025-02289-3 40221573 PMC12378151

[49] Zhu J Chen N Zhou M Guo J Zhu C Zhou J Calcium channel blockers versus other classes of drugs for hypertension. Cochrane Database Syst Rev. (2022) 1(1):CD003654. 10.1002/14651858.CD003654.pub6 35000192 PMC8742884

[50] Wang GM Li LJ Tang WL Wright JM. Renin inhibitors versus angiotensin converting enzyme (ACE) inhibitors for primary hypertension. Cochrane Database Syst Rev. (2020) 10(10):CD012569. 10.1002/14651858.CD012569.pub2 33089502 PMC8094968

[51] Lin DS- Wang T Buranakitjaroen P Chen C Cheng H Chia YC Angiotensin receptor neprilysin inhibitor as a novel antihypertensive drug: evidence from Asia and around the globe. J Clin Hypertens. (2021) 23(3):556–67. 10.1111/jch.14120 PMC8029571 33305531

[52] Neal B MacMahon S Chapman N, Blood Pressure Lowering Treatment Trialists’ Collaboration. Effects of ACE inhibitors, calcium antagonists, and other blood-pressure-lowering drugs: results of prospectively designed overviews of randomised trials. Blood pressure lowering treatment Trialists’ collaboration. Lancet. (2000) 356(9246):1955–64. 10.1016/S0140-6736(00)03307-9 11130523

[53] Wald DS Law M Morris JK Bestwick JP Wald NJ. Combination therapy versus monotherapy in reducing blood pressure: meta-analysis on 11,000 participants from 42 trials. Am J Med. (2009) 122(3):290–300. 10.1016/j.amjmed.2008.09.038 19272490

[54] Chiang C-E Wang T-D Ueng K-C Lin T-H Yeh H-I Chen C-Y 2015 guidelines of the Taiwan society of cardiology and the Taiwan hypertension society for the management of hypertension. J Chin Med Assoc. (2015) 78(1):1–47. 10.1016/j.jcma.2014.11.005 25547819

[55] Law MR Wald NJ Morris JK Jordan RE. Value of low dose combination treatment with blood pressure lowering drugs: analysis of 354 randomised trials. Br Med J. (2003) 326(7404):1427. 10.1136/bmj.326.7404.1427 12829555 PMC162261

[56] Wang N Rueter P Atkins E Webster R Huffman M de Silva A Efficacy and safety of low-dose triple and quadruple combination pills vs monotherapy, usual care, or placebo for the initial management of hypertension: a systematic review and meta-analysis. JAMA Cardiol. (2023) 8(6):606–11. 10.1001/jamacardio.2023.0720 37099314 PMC10134039

[57] Rodgers A Salam A Schutte AE Cushman WC de Silva HA Di Tanna GL Efficacy and safety of a novel low-dose triple single-pill combination of telmisartan, amlodipine and indapamide, compared with dual combinations for treatment of hypertension: a randomised, double-blind, active-controlled, international clinical trial. Lancet. (2024) 404(10462):1536–46. 10.1016/S0140-6736(24)01744-6 39426836

[58] Chow CK Atkins ER Hillis GS Nelson MR Reid CM Schlaich MP Initial treatment with a single pill containing quadruple combination of quarter doses of blood pressure medicines versus standard dose monotherapy in patients with hypertension (QUARTET): a phase 3, randomised, double-blind, active-controlled trial. Lancet. (2021) 398(10305):1043–52. 10.1016/S0140-6736(21)01922-X 34469767

[59] Huffman MD Baldridge AS Lazar D Abbas H Mejia J Flowers FM Efficacy and safety of a four-drug, quarter-dose treatment for hypertension: the QUARTET USA randomized trial. Hypertens Res. (2024) 47(6):1668–77. 10.1038/s41440-024-01658-y 38584159 PMC11150153

[60] Gupta AK Arshad S Poulter NR. Compliance, safety, and effectiveness of fixed-dose combinations of antihypertensive agents: a meta-analysis. Hypertension. (2010) 55(2):399–407. 10.1161/HYPERTENSIONAHA.109.139816 20026768

[61] Mallat SG Tanios BY Itani HS Lotfi T Akl EA. Free versus fixed combination antihypertensive therapy for essential arterial hypertension: a systematic review and meta-analysis. PLoS One. (2016) 11(8):e0161285. 10.1371/journal.pone.0161285 27548060 PMC4993355

[62] Wang N Salam A Webster R de Silva A Guggilla R Stepien S Association of low-dose triple combination therapy with therapeutic inertia and prescribing patterns in patients with hypertension: a secondary analysis of the TRIUMPH trial. JAMA Cardiol. (2020) 5(11):1219–26. 10.1001/jamacardio.2020.2739 32717045 PMC7376473

[63] Gnanenthiran SR Wang N Di Tanna GL Salam A Webster R de Silva HA Association of low-dose triple combination therapy vs usual care with time at target blood pressure: a secondary analysis of the TRIUMPH randomized clinical trial. JAMA Cardiol. (2022) 7(6):645–50. 10.1001/jamacardio.2022.0471 35416909 PMC9008553

[64] Mulè G Sorce A Carollo C Geraci G Cottone S. Self-blood pressure monitoring as a tool to increase hypertension awareness, adherence to antihypertensive therapy, and blood pressure control. J Clin Hypertens. (2019) 21(9):1305–7. 10.1111/jch.13638 PMC8030277 31393070

